# Annotations

**Published:** 1890-03-15

**Authors:** 


### Annotations.

**A New Danger from Tobacco.**

The influence of tobacco on human beings has, ever since its introduction, been a fruitful source of controversy. Burton, in his "Anatomy of Melancholy," gives expression to two views. Tobacco, divine, rare, excellent, which goes far beyond all the panaceas, potable gold, and philosophers'-stones, is a sovereign remedy in all diseases. But as abused, 'tis a plague, a mischief, a violent purge of goods, land, health, "hellish, devilish, damned tobacco, the ruin and overthrow of body and soul." The real meaning of which is, that tobacco in moderation is not injurious, while its excessive use causes many evils. Dr. E. Parkes summed up his experience and researches into the literature of tobacco smoking by saying: "I think we must decidedly admit injury from excess; from moderate use I can see no harm, except it may be in youth." The opponents of tobacco-smoking assert that its habitual use leads to decrease of bodily and mental vigour, and produces anæmia, palpitation and affections of the heart and circulation. It is also asserted that there is a disease of the eyes—tobacco amblyopia—caused by smoking. Yet it has been observed, that admitting all this, it is as nothing compared with the opposite results from the use of tobacco. The gentle exhilaration, the soothing and the social comfort derived from the "weed" commends it to one section of society; while the power it possesses of preventing waste of tissue, and, therefore, of appeasing the power of hunger, endears it to another section. Like many other things, it is susceptible of use and of abuse, and, like many other things, it cannot be indulged in equally by all. This is, however, no reason why we should follow the example of the Wahabees, and interdict the use of tobacco altogether, on penalty of the loss of a hand to those transgressing! Unless, indeed, the following is correct. It is stated that a German physician, on examination of a number of cigar tips, found that many of them were infected with tubercle bacilli! The makers were tuberculous, and in the manufacture of the cigars moistened the tips with their saliva! This certainly represents a new danger from using tobacco, at least, in the shape of cigarettes and cigars. We were aware that there is considerable difference between bad and good tobacco, and have been inclined to attribute injurious effects to the use of inferior produce. Any tobacco, however, may be impregnated as above. And if tobacco may be contaminated by one bacillus, there does not appear any reason why it should not harbour other microbes. This is a point which will doubtless be taken up by the anti-tobacconists, and if it reduces the consumption of tobacco it will not do any harm except to the revenue.

**A New Pain Killer.**

The public, whilst divided in opinion as to the value of vivisection operations, is always interested in experiments with drugs that promise to relieve pain or to cure disease. So far as doctors and their science are concerned, every non-medical person is a pure utilitarian. During last year two French physicians, Dr. Dujardin-Beaumetz and Dr. Bardet investigated the therapeutic activities of Exalgine, a substance similar in composition to Antipyrin and Antifebrin. Exalgine has proved to be very energetic in its action as a pain killer. Neuralgias of all kinds, whether in the head, face, chest, legs, or elsewhere, are generally dispelled by sufficient doses in about half-an-hour. Very frequently the pain does not return and the cure is permanent. Sometimes, however, there may be a relapse in the course of a few hours, and further doses are required. This is one side of the question, and no doubt many persons who are suffering from distressing neuralgia would be very glad to be informed as to the dose of the drug and the method of using it. We think, however, that we shall better serve the interests of our readers by giving them a word of caution. Exalgine has the unfortunate property of being a powerful poison as well as a pain killer. Quite small doses will kill rabbits with remarkable promptitude. The poison affects the brain, and gives rise to violent convulsions like those of epilepsy. Between the convulsive attacks there are intervals of quiescence, during which the animal lies trembling, panting, and almost suffocated. These are the outward appearances of which the non-medical mind can take account. Changes also take place in the blood and in the action of the heart of such a kind as to make medical men employ the new remedy with extreme caution. It is more than remarkable how rashly some persons will deal with substances which are more dangerous than edged tools in a child's hands. A cautious medical man—and every doctor ought to be an embodiment of caution—will watch carefully for years the action of a new drug before he can feel quite assured that it will not play one or other of his patients some unexpected trick. But the lay-man or lay-woman, when in racking pain, will cry out for anything in the world to be given if only the pain can be taken away. Men and women forget what infinitely complicated creatures they are. One man's brain may be unable to bear the use of a particular drug; another man's heart may be the defective organ; the liver of a third may be at fault; the kidneys of a fourth, the stomach of a fifth, the lungs of a sixth, and so on. There is no doubt that the drugs used by the modern doctor are much more potent than those that were in use even half a-century ago. But most of these drugs are dangerous in the exact degree of their potency, and the skill required in their administration is to be measured by their dangerousness. Our counsel with regard to the new remedy is, that it should on no account be tasted, even in small quantities, except under competent professional direction.

**The Princess of Wales and Nurses' Pensions.**

The Princess of Wales, it was stated at the annual general meeting of the Pension Fund, has agreed to sign the official certificates and to present them to the first thousand nurses who joined the Society. Whoever was the first to think of asking her Royal Highness' signature for such a purpose must be credited with a happy inspiration. That the Princess should have granted both requests shows a womanly sympathy with nurses and their work, which is honourable alike to her judgment and her heart. The first thousand nurses, who in the teeth of much adverse criticism, showed their faith in the Pension Fund and its managers by venturing themselves and their money, will, it appears, have a considerable number of privileges, large and small. Not the least will be the fact of their having entered into a life-long covenant with so noble a woman as their future Queen. The financial success of the Pension Fund is one of the most remarkable business phenomena of the times. According to the annual report, the invested funds on the last day of 1889 amounted to £51,212 7s. 10d. At the same period the Bonus Fund alone stood at more than thirty-three thousand pounds. At the present time it amounts to thirty-eight thousand. When it is remembered that the Pension Fund is not yet three years old, that the Bonus Fund has been in existence little more than one year, and that no appeal of any kind has been made to the public at large, it must be evident to the simplest capacity that a future is being prepared for trained nurses which will, in some measure, show the universal appreciation of their noble, devoted, and invaluable work. No doubt nurses are women, and being women there may be found among them persons of little feeling and less worth. But our experience of them, gained from almost twenty years of medical practice, has established in us the conviction that the average nurse is not an ordinary woman either in tenderness of feeling, in devotion to duty, or in natural and acquired capacity. Nurses, as a class, represent the best of womankind. The instinctive sympathy with suffering and sorrow that makes a woman desire to enter the nursing profession, and the steadfastness of will that carries her through the arduous, and sometimes repulsive, duties of her training, prove beyond doubt our contention that the sick nurse is made of worthier stuff than the average woman. The writer gives utterance, not only to his own feelings, but to those of every right-minded person who has experience of the subject, when he expresses profound gratitude to the Princess of Wales and also to the Prince for their practical interest in nurses and sympathy with their Pension Fund. The donors, too, who have in so short a time subscribed so generous a sum as nearly forty thousand pounds deserve the deepest gratitude both of the nurses and all who are in any way associated with them. The Pension Fund now floats in the calm waters of assured prosperity. It is destined to prove an ark of safety for many a storm-tossed and weary, but true and noble, woman.

